# Identification of potential target genes of USP22 via ChIP-seq and RNA-seq analysis in HeLa cells

**DOI:** 10.1590/1678-4685-GMB-2017-0164

**Published:** 2018-06-11

**Authors:** Zhen Gong, Jianyun Liu, Xin Xie, Xiaoyuan Xu, Ping Wu, Huimin Li, Yaqin Wang, Weidong Li, Jianjun Xiong

**Affiliations:** 1 Jiujiang University Jiujiang University College of Basic Medical Science JiujiangJiangxi China College of Basic Medical Science, Jiujiang University, Jiujiang, Jiangxi, China; 2 Jiujiang University Jiujiang University Key Laboratory of Jiangxi Province for the Systemic Bio-medicine JiujiangJiangxi China Key Laboratory of Jiangxi Province for the Systemic Bio-medicine, Jiujiang University, Jiujiang, Jiangxi, China; 3 Wuhan University Wuhan University Renmin Hospital Reproductive Medical Center WuhanHubei Province China Reproductive Medical Center, Renmin Hospital of Wuhan University, Wuhan, Hubei Province, China

**Keywords:** USP22, target genes, ChIP-seq, knockdown, RNA-seq

## Abstract

The ubiquitin-specific protease 22 (USP22) is an oncogene and its expression is upregulated in many types of cancer. In the nucleus, USP22 functions as one subunit of the SAGA to regulate gene transcription. However, the genome-wide USP22 binding sites and its direct target genes are yet clear. In this study, we characterized the potential genomic binding sites of UPS22 and GCN5 by ChIP-seq using specific antibodies in HeLa cells. There were 408 overlapping putative target genes bound by both USP22 and GCN5. Motif analysis showed that the sequences bound by USP22 and GCN5 shared two common motifs. Gene ontology (GO) and pathway analysis indicated that the genes targeted by USP22 and GCN5 were involved in different physiological processes and pathways. Further RNA-seq, GO and pathway analyses revealed that knockdown of UPS22 induced differential expression of many genes that participated in diverse physiological processes, such as metabolic process. Integration of ChIP-seq and RNA-seq data revealed that UPS22 bound to the promoters of 56 genes. These findings may provide new insights into the regulation of USP22 on gene expression during the development of cervical cancer.

## Introduction

Ubiquitin-Specific Peptidase 22 (USP22) belongs to the largest subfamily of ubiquitin-specific proteases (USPs). In human tissues, USP22 is expressed moderately in the heart and skeletal muscle, and weakly in the lung and liver. In mouse tissues, *Usp22* is expressed strongly in the brain and dynamically expressed during the early embryonic development (Lee *et al.*, 2006). Recently, USP22 over-expression is found in several types of cancers and associated with the recurrence, metastasis and poor survival of patients with cancers (Piao *et al.*, 2012; Tang *et al.*, 2015). Actually, USP22 has been identified as one of the putative cancer stem cell markers (Glinsky, 2005; Glinsky, 2006).

Functionally, USP22 removes ubiquitin from the target protein by its catalytic domain at the C-terminal. There are two kinds of proteins identified as the substrates of USP22, non-histone and histone. It is well known that USP22 can interact with non-histone substrates to stabilize these proteins and inhibit their degradation by proteasome. These substrates include telomeric repeat binding factor 1 (TRF1) (Atanassov *et al.*, 2009), sirtuin 1 (SIRT1) (Lin *et al.*, 2012), cyclooxygenase 2 (COX-2) (Xiao *et al.*, 2015), lysine-specific histone demethylase 1A (KDM1A) (Zhou *et al.*, 2016) and others. Furthermore, USP22 is the subunit of the transcription regulatory SAGA (Spt-Ada-Gcn5 acetyltransferase) complex (Zhao *et al.*, 2008; Zhang *et al.*, 2008). As a multi-subunit complex, SAGA is organized by several functional submodules: the deubiquitinating module (DUBm), the histone acetyltransferase (HAT) module, and the SPT and TAF modules. USP22 and GCN5 are the essential proteins linked to DUB module and HAT module in human SAGA, respectively. Through modifying histone H2A and H2B, USP22 plays a key role in facilitating a number of cellular events, including gene regulation. Therefore, up-regulation of USP22 expression will lead to abnormal activation of multiple pathways to promote cell survival while down-regulation of USP22 expression can induce cell cycle arrest at G0/G1 phase in different types of cancer cells (Zhang *et al.*, 2008). However, there is no information on the genome-wide binding sites of USP22 and its direct target genes in cancer cells.

In this study, we employed chromatin immunoprecipitation sequencing (ChIP-seq) technology to study the potential targets of USP22 in human cervical cancer cells. Furthermore, we explored transcriptome profiling in response to USP22 silencing. Our data may provide new insights into the role of USP22 in regulating the expression of genes associated with cancer progression.

## Materials and Methods

### Cell culture and ChIP

HeLa cells were grown in DMEM medium supplemented with 10% fetal bovine serum (FBS). ChIP assays were performed using the EZ-ChIP Kit (Millipore), according to the manufacturer instructions. Briefly, when the cells reached at 80% confluency, the cells were cross-linked with 1% of formaldehyde in culture medium at room temperature for 10 min, which was quenched by glycine solution. The cells were harvested and suspended in 1% SDS Lysis Buffer for 10 min. The cell lysates were sonicated to breakdown cellular DNA into an average length of 500 bp and centrifuged at 10,000 x *g* for 10 min to remove cellular debris.

The cell lysates were reacted with protein G agarose beads to preclear the chromatin at 4 °C for 1 h with rotation. After brief centrifugation and protein quantification, the supernatants were reacted with anti-USP22 (2 μg), anti-GCN5 (2 μg) or negative control IgG (2 μg, Santa Cruz Biotechnology, Santa Cruz, USA) for 12 h with rotation. The formed immunocomplex in the samples was precipitated with protein G agarose beads for 1 h and centrifuged, followed by washed with cold buffers. The bound DNA/antigen/antibody complex was eluted with 100 μL of elution buffer and incubated at 65 °C for 12 h. Subsequently, the samples were treated with RNase A (10 μg/μL) at 37 °C for 2 h and then with proteinase K (20 μg/ml) at 55 °C for 2 h. Finally, the contained DNA was purified by spin columns. Similarly, the input genomic DNA was obtained through the elution and purification procedures. All DNA samples were quantified using NanoDrop 2000 spectrophotometer.

### ChIP-seq

ChIP-seq libraries were generated for pair-end sequencing using the TruSeq DNA LT Sample Prep Kit (Illumina, San Diego, CA), according to the manufacturer’s instructions. Briefly, the fragmented DNA samples (1 μg/each, in duplicate) were end-repaired, A-tailed at the 3’end and ligated with indexed adapters provided. The potential target DNA samples were extracted using AMPure XP magnetic beads and amplified by PCR to create the final ChIP-seq libraries, which were quantified by Agilent 2200. The DNA in the ChIP-seq libraries was sequenced twice in the Solexa sequencer (PE150), according to the manufacturer’s instructions (Illumina).

### ChIP-PCR

ChIP DNA was analyzed by quantitative PCR using the SYBR Green supermix and specific primers in a 7300 sequence detection system (Applied Biosystems, Foster City, CA). The primers were designed to cover regions that were sequenced in the ChIP-seq experiment and are shown in Table S1. The PCR reactions were performed in triplicate at 95°C for 1 min and subjected to 40 cycles of 95°C for 15 s and 60°C for 34 s, followed by 72°C for 6 min. The relative levels of DNA expression were calculated by the 2^-^ΔΔCT method. The GAPDH promoter regions were used as negative controls for USP22 and GCN5 binding.

### RNA interference

HeLa cells were infected with USP22-specific shRNA lentiviral particles (sc-63195-V, Santa Cruz Biotechnology) or control shRNA lentiviral particles (sc-108080, Santa Cruz Biotechnology), following the manufacturer’s recommendations. Briefly, the cells (5 x 10^5^) were cultured in complete optimal medium in a 25 cm^2^ flask overnight and infected with 5 μL of shRNA lentiviral particles in 2 mL of 5 μg/mL polybrene media mixture for 12 h, followed by changing to complete optimal medium. Three days after infection, the cells were harvested and subjected to western blot analysis and RNA-seq.

### Western blot

The different groups of cells were harvested and lysed in the lysis buffer, followed by centrifugation. After being quantified with BCA reagents, the cell lysates (30 μg/lane) were separated by sodium dodecyl sulfate polyacrylamide gel electrophoresis (SDS-PAGE) on 10% gels and transferred on polyvinylidene difluoride (PVDF)membranes. The membranes were blocked with 5% fat-free dry milk in TBST and incubated with anti-USP22 or anti-GAPDH overnight at 4 ºC. The bound antibodies were detected by horseradish peroxidase(HRP)-conjugated second antibodies and visualized using the enhanced chemiluminescence. The relative levels of USP22 to control GAPDH were determined by densitometric analysis using the ImageJ software.

### RNA-seq

Total RNA was extracted from the cells using Trizol reagent (Invitrogen). The quality and quantity of each RNA sample were measured using Bioanalyzer 2200 (Agilent) and the RNA samples were kept at -80 ºC. An RNA sample with a RIN >8.0 was used for rRNA depletion.

The cDNA libraries of each pooled RNA sample for single-end sequencing were generated using the Ion Total RNA-Seq Kit v2.0 (Life Technologies, Carlsbad, CA), according to the manufacturer’s instructions. Briefly, the contaminated rRNA in RNA samples were depleted and the RNA was fragmented into 150-200 bp using divalent cations at 94 ºC for 8 min. The cleaved RNA fragments were reversely transcribed into cDNA, which were end-repaired, A-tailed at the 3’end and ligated with indexed adapters provided. The potential target DNAs were extracted using Nucleic Acid Binding Beads, purified and amplified by PCR to create the final cDNA libraries, followed by quantification using Agilent 2200.

The cDNA libraries were subjected to the Proton Sequencing device, according to commercially available protocols. Briefly, the samples were diluted and mixed. The mixture was processed on a OneTouch 2 instrument (Life Technologies) and enriched on a OneTouch 2 ES station (Life Technologies) for preparing the template-positive Ion PI Ion Sphere Particles (Life Technologies) according to the Ion PI Template OT2 200 Kit v2.0 (Life Technologies). After enrichment, the mixed template-positive Ion PI Ion Sphere Particles in individual samples were loaded on to 1 P1v2 Proton Chip (Life Technologies) and sequenced on the Proton Sequencer, according to the Ion PI Sequencing 200 Kit v2.0 (Life Technologies) by NovelBio Laboratory, Shanghai. The changed RNAs were validated by quantitative PCR using the primers listed in Table S2.

### Data analysis

Clean reads were obtained from the raw reads after removing the adaptor sequences, reads with >5% ambiguous bases (noted as N) and low-quality reads containing more than 20 percent of bases with qualities of <13. The raw sequencing data were evaluated by FAST-QC, including quality distribution of nucleotides, position specific sequencing quality, GC content, the proportion of PCR duplication, and kmer frequency.

Clean reads from the genomic sequencing were aligned to the human reference genome sequence GRCH38.p2 using Bowtie2(v2.0.5) (Langmead *et al.*, 2012). Transcriptional start site (TSS) and chromosomal distribution were obtained by custom Java scripts. The potential genes were defined within +5 kilobases (kb) from the TSS, and 50 kb downstream from the transcription end site (TES). The clean reads from RNA-seq were aligned to the human reference genome sequence GRCH38.p2 using the MapSplice program (v2.1.6) (Wang *et al.*, 2010), which can identify the exon-exon splicing immediately and accurately. The experimental data were first optimized for the alignment parameters to provide the largest information on the AS events. The potential genes sequenced were counted by HTseq and their relative expression levels were determined by RPKM method (Anders *et al.*, 2015).

The differentially expressed genes were identified using the DEseq algorithm, according to both fold change (>1.5 or <0.67) with a false discovery rate (FDR, <0.05) and threshold 4 (Kallio *et al.*, 2011). Regions enriched in the genome were determined using MACS (v1.4.1). Peak statistics and annotation were analyzed using custom java scripts. Peak associated genes were selected for downstream gene ontology (GO) and pathway analysis. The GO analysis was performed to elucidate the biological implications of unique genes in the significant or representative profiles of the selected genes or differentially expressed genes (Ashburner *et al.*, 2000). The GO annotations were integrated from NCBI (http://www.ncbi.nlm.nih.gov/), UniProt (http://www.uniprot.org/) and the GO (http://www.geneontology.org/). The significant GO categories were analyzed by Fisher’s exact test and χ^2^ test and the p-values were corrected by FDR.

The significant pathway of the selected genes in the experiment was analyzed by the Fisher’s exact test, according to KEGG database. The threshold of significance was defined by P-value and FDR (Draghici *et al.*, 2007).

## Results

### Occupancy of USP22 and GCN5 at gene loci

**Figure 1:**
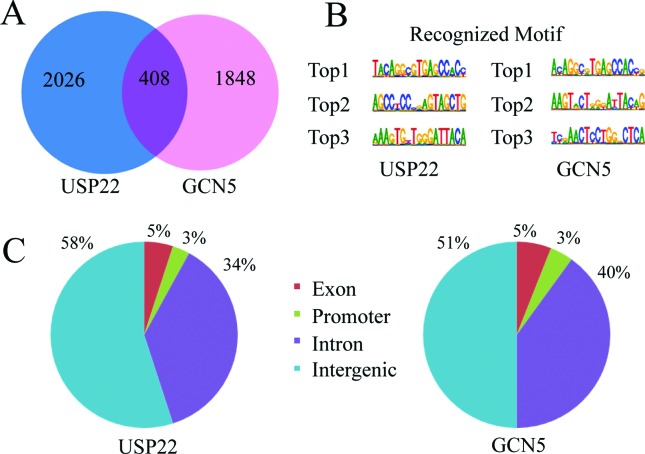
Characterization of USP22 and GCN5 binding sites in human genome. (A) Total number of genes with USP22 and GCN5 occupancy in two independent ChIP samples. There were 408 gene targets bound by both USP22 and GCN5. (B) Motif analysis of USP22 or GCN5-bound genomic regions showing the top 3 enriched new sequence motifs. (C) Pie charts show the distribution of USP22 or GCN5 binding sites in the genome.

To identify the potential gene targets of USP22 and GCN5, the genomic DNA fragments recognized by USP22 and GCN5 were isolated by ChIP using specific antibodies and sequenced. Analysis of the ChIP-seq data revealed that there were total 2.52 million short reads from the USP22 immunoprecipitated samples and 2.45 million short reads from the GCN5 immunoprecipitated samples. Among short reads, 1.74 million USP22 ChIP-Seq reads and 1.68 million GCN5 ChIP-Seq reads were aligned to the human reference genome using Bowtie2(v2.0.5). The sequence length was 35 bp. To identify and annotate the target genes, we introduced the MACS for peak calling filtered with P-value and peak enrichment and domestic java code to annotate the peak regions in gene promoter regions following the promoter regions ranged from 0 bp ~ -2000 bp (the standard definition). There were 2434 putative target genes bound by USP22 (Table S3), including the known target *MTA 1* and *CAD* (Zhang *et al.*, 2008), and 2256 putative target genes bound by GCN5 (Table S4), respectively. Of these genes, 408 genes were bound potentially by both USP22 and GCN5, which accounted of 16.8% of genes recognized by USP22 and 18.1% recognized by GCN5 (Figure 1A).

Next, we characterized the potential motifs in the target sequences using XX motif software. We found that the top 3 frequent new sequences bound by USP22 were TACAGGCGTGAGCCAC, AGCC(T/C)CCCGAGTAG CTG and AAAGTG(T/C)TGGGATTACA; whereas by GCN5 were A(C/T)AGGC(G/A)TGAGCCAC(C/T), AAGT(A/G)CT(G/A)(G/A)(G/T)A(T/C)TAC(A/T)G, T(C/T)(C/A)AACTCCTGGCTCA. Interestingly, the most frequent motif of USP22 binding was perfectly matched with GCN5. Furthermore, the third highest frequent motif of USP22 binding had 90% similarity with the second highest frequent motif of GCN5 binding (Figure 1B). In addition to these motifs, there was no similar motif between USP22 and GCN5 binding sites.

The USP22 and GCN5 binding sites had similar distributions across the whole genome. Further analysis showed that the majority of USP22 binding sites was located within intronic and intergenic regions, and approximately 58% of the peaks were located in intergenic, 34% in introns, 5% in exons and 3% in the promoter. For GCN5 51% of the binding sites were in intergenic, 40% in introns, 5% in exons and 4% in the promoter (Figure 1C).

### Characterization of the putative target genes of USP22 and GCN5

**Figure 2:**
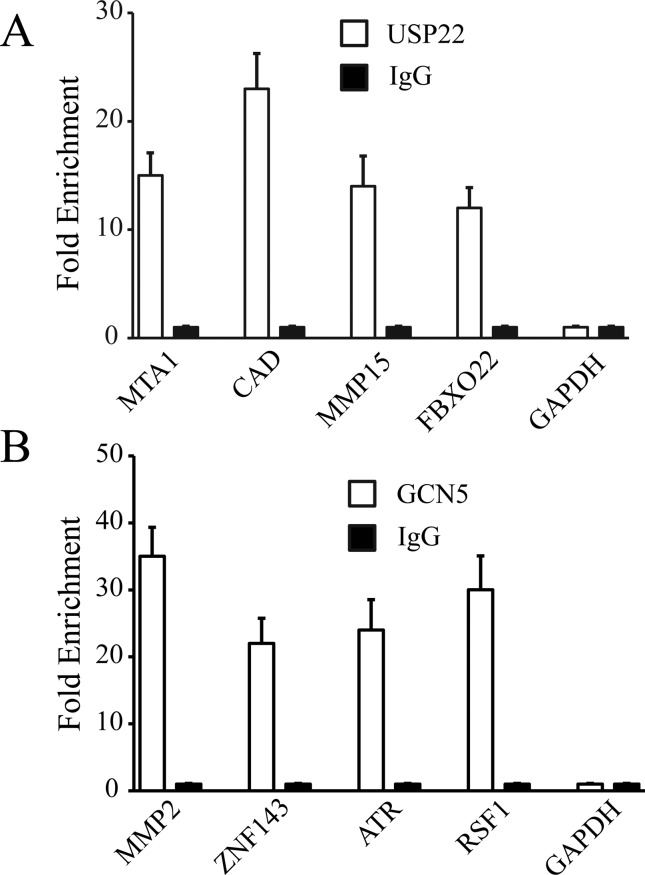
ChIP-PCR analysis of the selected USP22 or GCN5 binding sites. ChIP-qPCR was used to amplify chromatin derived from immunoprecipitations with (A) anti-USP22 antibody or (B) anti-GCN5 antibody as indicated. The levels of control IgG bound genes were designated as 1. Each data point represents the average of two independent ChIP experiments.

We validated four binding sites selected randomly for USP22 or GCN5, respectively. The USP22 putative target genes were *MTA1*, *CAD*, *MMP15*, and *FBXO22*. The GCN5 putative target genes were *MMP2*, *ZNF143*, *ATR*, and *RSF1*. Quantitative PCR analysis showed that all the selected genes, but not the negative control GAPDH, were significantly enriched by ChIP, as compared to the input group (Figure 2), validating the efficacy of ChIP.

### Gene ontology analysis and KEGG pathway analysis

**Figure 3:**
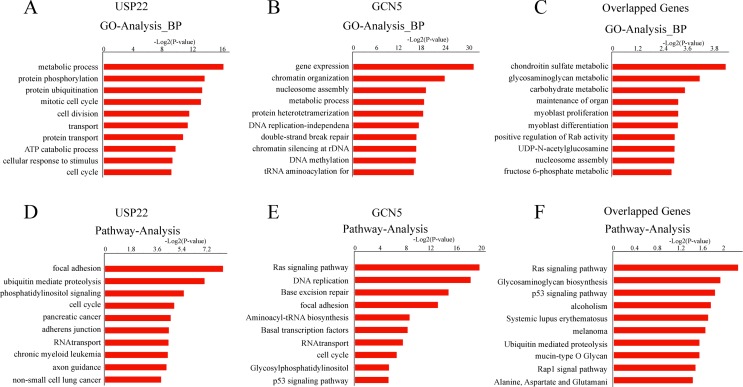
GO category and KEGG pathway analysis of the target genes bound by UPS22 and GCN5 in HeLa cells. (A) GO categories for USP22 binding genes; (B) GO categories for GCN5 binding genes; (C) GO categories for both USP22 and GCN5 binding genes; (D) Pathway analysis of USP22 binding genes; (E.) Pathway analysis of GCN5 binding genes; (F) Pathway analysis of both USP22 and GCN5 binding genes.

The genes recognized by USP22 or GCN5 were classified into different functional categories by GO analysis. The genes recognized by USP22 were involved in diverse physiological processes, such as metabolic (GO:0008152), protein phosphorylation (GO:0006468), protein ubiquitination (GO:0016567), mitotic cell cycle (GO:0000278), and others (Figure 3A). The genes bound by GCN5 participated in gene expression (GO: 0010467), chromatin organization (GO: 0006325), nucleosome assembly (GO: 0006334), metabolic process (GO: 0008152), and others (Figure 3B). The genes targeted by both USP22 and GCN5 mainly focused on metabolic process, such as the chondroitin sulfate metabolic process (GO: 0030204), glycosaminoglycan metabolic process (GO: 0030203), and carbohydrate metabolic process (GO: 0005975) (Figure 3C).

Pathway analysis indicated that the genes targeted by USP22 and GCN5 were involved in different pathways. The genes targeted by USP22 were involved in focal adhesion, ubiquitin-mediated proteolysis, phosphatidylinositol signaling, cell cycle and others, whereas GCN5 recognized genes that participated in Ras signaling, DNA replication, base excision repair, focal adhesion and others (Figure 3D,E). The genes targeted by both USP22 and GCN5 regulated the Ras signaling, glycosaminoglycan biosynthesis, p53 signaling, and others (Figure 3F).

### RNA-seq and combined with ChIP-seq data

**Figure 4:**
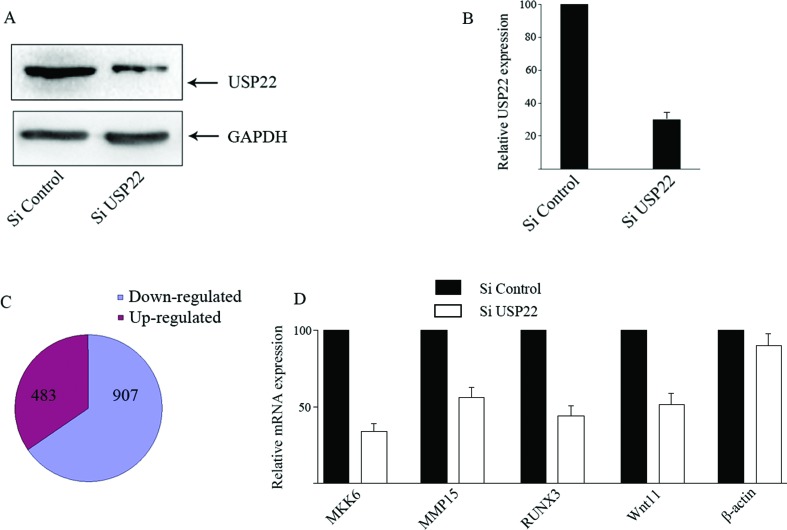
Knockdown of UPS22 by siRNA modulates gene expression in HeLa cells. (A and B) Western blot analysis of USP22 silencing in HeLa cells. (C) Pie charts show the ratio of up-regulated or down-regulated genes by UPS22 silencing in HeLa cells. (D) Quantitative RT-PCR analysis of the relative levels of mRNA transcripts of some genes targeted by UPS22. Data are expressed as the mean ± SD of each group of genes and the levels of mRNA transcripts in the control siRNA-transfected cells were designated as 100. Data are representative of three independent experiments.

To further investigate the regulation of target gene expression by USP22, HeLa cells were infected with control lentivirus or lentivirus for USP22-specific shRNA expression. Western blot analysis indicated that infection with the lentivirus for UPS22-specifc shRNA reduced the relative levels of USP22 expression by near 75% (Figure 4A,B). RNA-seq analysis identified that the relative levels of 1,390 mRNA transcripts were altered by at least 1.5-fold (Table S5). There were 907 down-regulated genes and 483 up-regulated ones in the UPS22-silenced cells (Figure 4C). Further RT-PCR analysis revealed that the relative levels of MKK6, MMP15, WNT11 and RUNX3 mRNA transcripts, but not the control β-actin, were significantly reduced in the UPS22-silenced cells, as compared with that in the control cells (Figure 4D).

**Figure 5:**
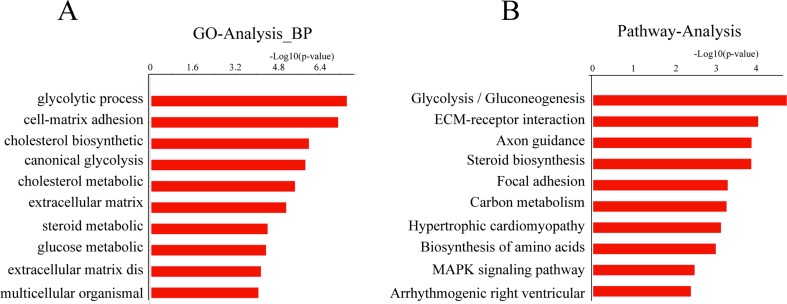
GO category and KEGG pathway analysis of down-regulated genes in UPS22 silencing HeLa cells. (A) The significant GO categories for down-regulated genes using the threshold of P < 0.05 and FDR < 0.05 for the selection of significant GO categories. (B) The significant pathways of down-regulated genes using the threshold of P < 0.05 and FDR < 0.05 for the selection of significant KEGG pathways.

In addition, the potential function of differentially expressed genes targeted by UPS22 were analyzed by GO and pathway analyses. The results showed that the down-regulated genes were involved in glycolytic process (GO: 0006096), cell-matrix adhesion (GO: 0007160), cholesterol metabolic process (GO: 0008203). The KEGG pathway analysis indicated that down-regulated genes participated in glycolysis/gluconeogenesis, ECM-receptor interaction, focal adhesion and others (Figure 5).

To determine whether the differentially expressed genes induced by UPS22 silencing were also bound by USP22, we integrated the ChIP-seq and RNA-seq datasets and compared the expression of genes with a USP22 binding site. Among the 1,390 differentially expressed genes, only 345 genes were bound by USP22. Unexpectedly, only 56 genes were bound by USP22 at the promoter region.

## Discussion

Up-regulation of USP22 expression is associated with the development and progression of several types of cancers and leads to abnormal activation of multiple pathways that support cell survival (Schrecengost *et al.*, 2014). Given its gene regulation function, identification of the genomic binding sites of UPS22 is important for understanding its role in the development of cancer. Unlike a transcription factor, USP22 does not contain a classic DNA binding domain. In eukaryotes, USP22 is one component of the SAGA transcriptional cofactor complex to regulate gene expression (Zhang *et al.*, 2008; Pijnappel *et al.*, 2008). Besides USP22, the SAGA complex contains several other proteins, including GCN5, SPT3 or TAF10 (Koutelou *et al.*, 2010). In this study, we characterized the potential binding sites of UPS22 and GCN5 by ChIP. We found either USP22 antibody or GCN5 antibody precipitated a large number of DNA fragments, indicating that either USP22 or GCN5 widely interacted with different genes in the genome of HeLa cells. Furthermore, the USP22 and GCN5 binding sites were widely distributed in intergenic and intronic regions across the whole genome, but a few in the promoter region. These suggest that USP22 and GCN5 may function not specifically in the promoter region and they may regulate genes from sites that are distant from the proximal promoter regions in HeLa cells.

MACS analysis indicated that there were many genes potentially bound by USP22 or GCN5. The USP22 bound genes included the known target genes of the *MTM* and *CAD*. However, there are only a small part of genes bound by both USP22 and GCN5, indicating that the target genes regulated by USP22 and GCN5 were not completely the same. This suggests that USP22 and GCN5 may not function specifically in the SAGA complex and they may correlate with different cofactors in addition to the SAGA. Actually, GCN5 has been found as one component in the transcription complex ATAC, which is different from the SAGA and regulates target genes that are distinct from those of the SAGA (Wang *et al.*, 2008; Guelman *et al.*, 2009). This also explains that USP22 or GCN5 bound to different sites in the genome. Alternatively, USP22 may also exit in another transcription complex or interact with other transcription factors to regulate genes transcription.

Consistent with the varying gene binding sites, the motif analysis revealed that the top 3 frequent new motifs recognized by USP22 shared with GCN5. Similarly, two out of three highest motifs recognized by GCN5 were also bound by USP22. Theses results suggest that USP22 and GCN5 may not only share the common DNA consensus, but also have each specific binding preference. We are interested in further determining which cofactors coordinate with USP22 to recognize these novel motifs.

GO analysis of the ChIP-seq data revealed the USP22 or GCN5 could regulate diverse physiological processes. Notably, there were 408 overlapping genes involved in metabolic processes, suggesting their potentially important functions in regulation of metabolism. Pathway analysis of USP22 or GCN5 target genes indicated that they regulated multiple regulatory networks. In addition to focal adhesion and cell cycle, USP22 also controlled the pathways related to ubiquitin-mediated proteolysis and phosphatidylinositol signaling, which were different from GCN5.

Furthermore, knockdown of USP22 modulated the expression of many genes in HeLa cells. Although UPS22 may not directly regulate these gene expression USP22 may indirectly regulate some gene expression in HeLa cells. In addition, integration of the RNA-seq and ChIP-seq data revealed that some genes down-regulated by UPS22 silencing were directly bound by USP22. Unexpectedly, UPS22 only bound to the promoters of a few of the genes regulated by USP22. These support the notion that the genes tightly bound by an activator are not necessarily the most responsive to modulations in the transcription factor level (van der Deen *et al.*, 2012). It is also unlikely that this is may be attributed to incomplete down-regulation of USP22 expression in HeLa cells in our experimental system. Alternatively, many binding sites by UPS22 may not be functional.

Further GO and pathway analysis indicated that some genes down-regulated by UPS22 silencing are involved in metabolic process and regulation. This suggests that USP22 may regulate metabolic processes, consistent with ChIP-seq GO analysis of genomic targets by UPS22. Furthermore, the knockdown of UPS22 also changed the levels of genes for cellular matrix and adhesion, supporting that UPS22 promotes cancer progression.

In conclusion, we used the ChIP-seq and RNA-seq technologies to analyze the binding sites of USP22 and its potential target genes in HeLa cells. These findings may provide new insights in understanding the role of USP22 in the development and progression of cervical cancer.

## Supplementary material

The following online material is available for this article:

Table S1 - Primers for validation of ChIP-target genes.

Table S2 - Primers for validation of down-regulated genes.

Table S3 - Target genes in USP22 ChIP-seq.

Table S4 - Target genes in GCN5 ChIP-seq.

Table S5 - Target genes in USP22 RNA-seq.
