# Supplementary material for: Identification of potential target genes of USP22 via ChIP-seq and RNA-seq analysis in HeLa cells
Source: Genet Mol Biol. 2018 Jun 11;41(2):488–95. doi: 10.1590/1678-4685-GMB-2017-0164 (PMC6082230; doi:10.1590/1678-4685-GMB-2017-0164)
Supplement: Supplementary file 1 [file 1415-4757-GMB-1678-4685-GMB-2017-0164-s001.pdf]

Supplementary Material to “Identification of potential target genes of USP22 via ChIP-seq and RNA-seq analysis in HeLa cells”

Table S1 - Primers for validation of ChIP-target genes

Gene	Forward primer	Reverse primer
MTA1	ACGCTCCGCTCCATGTCCTA	TCCAGTGAGTGACCCAGACG
MMP15	GGTGAGGACGGCTTCCATTT	GGCTGGTGCGAGTGAAGTGC
FBXO22	CAGGTGAATCTGATGTCCCT	TGCTCTTCGTAGTCCCAATG
CAD	TAGGCAGTGGAGTTAGGAG	CCACCATTGTGATTTGTTT
ZNF143	AACATCATCCAGCTTTCTCC	CAGCCCTTTCTAAACCTTTG
MMP2	AAACTGTTCCCTGCTGACCC	AAGAGGTGGAGTGCTGGGTG
ATR	AAGAGGGTGAGACAGAATG	GGAAAGATGACCACCATAA
RSF1	GTACTCACTATGTGCTACGG	AGTTTGTTAGAAGACAGGGTT
GAPDH	TGCCTTGCTCTTGCTACTCT	GTTTAGCCTGCCTGGTGATA
