# Supplementary material for: Identification of potential target genes of USP22 via ChIP-seq and RNA-seq analysis in HeLa cells
Source: Genet Mol Biol. 2018 Jun 11;41(2):488–95. doi: 10.1590/1678-4685-GMB-2017-0164 (PMC6082230; doi:10.1590/1678-4685-GMB-2017-0164)
Supplement: Supplementary file 2 [file 1415-4757-GMB-1678-4685-GMB-2017-0164-s002.pdf]

Supplementary Material to "Identification of potential target genes of USP22 via ChIP-seq and RNA-seq analysis in HeLa cells"

Table S2 - Primers for validation of down-regulation genes

Gene	Forward primer	Reverse primer
MKK6	ACGCTCCGCTCCATGTCCTA	TCCAGTGAGTGACCCAGACG
MMP15	GGTGAGGACGGCTTCCATTT	GGCTGGTGCGAGTGAAGTGC
RUNX3	CAGGTGAATCTGATGTCCCT	TGCTCTTCGTAGTCCCAATG
WNT11	TGACCTCAAGACCCGATACCT	CGAGTTCCGAGTCCTTCACA
β-actin	GCGAGAAGATGACCCAGATCATGT	TACCCCTCGTAGATGGGCACA
